# One-Stop Surgery: An Innovation to Limit Hospital Visits in Children

**DOI:** 10.1055/s-0041-1740158

**Published:** 2021-12-02

**Authors:** Kelly M.A. Dreuning, Joep P.M. Derikx, Ayoub Ouali, Liedewij M.J. Janssen, Maurits W. van Tulder, Jos W.R. Twisk, Lotte Haverman, L.W. Ernest van Heurn

**Affiliations:** 1 Department of Paediatric Surgery, Emma Children's Hospital, Amsterdam UMC, University of Amsterdam & Vrije Universiteit Amsterdam, Amsterdam Reproduction and Development Research Institute, and the Amsterdam Public Health Research Institute, Amsterdam, the Netherlands; 2 Department of Medical Informatics, University of Amsterdam, Amsterdam, the Netherlands; 3 Department of Anesthesiology, Amsterdam UMC, Vrije Universiteit Amsterdam, Amsterdam, the Netherlands; 4 Department of Health Sciences, Amsterdam Movement Science Research Institute, Faculty of Science, Vrije Universiteit Amsterdam, Amsterdam, the Netherlands; 5 Department of Physiotherapy & Occupational Therapy, Aarhus University Hospital, Aarhus, Denmark; 6 Department of Methodology and Applied Biostatistics, Vrije Universiteit Amsterdam, Amsterdam, the Netherlands; 7 Psychosocial Department, Emma Children's Hospital, Amsterdam UMC, University of Amsterdam, Amsterdam, the Netherlands

**Keywords:** child, inguinal hernia, hernia repair, one-stop surgery

## Abstract

**Introduction** One-stop surgery (OSS) allows for same-day outpatient clinic visit, preoperative assessment, and surgical repair. This study aims to determine the efficiency, (cost-)effectiveness, and family satisfaction of one-stop inguinal hernia surgery compared with usual care.

**Material and Methods** Children (≥ 3 months) with inguinal hernia and American Society of Anesthesiologists (ASA) grades I–II, scheduled for OSS (intervention) or regular treatment (control) between March 1, 2017, and December 1, 2018, were eligible for inclusion. Exclusion criteria consisted of age less than 3 months and ASA grades III–IV. The primary outcome measure was treatment efficiency (i.e., total number of hospital visits and waiting time [days] between referral and surgery). Secondary outcome measures were the effectiveness in terms of complication and recurrence rate, and parent-reported satisfaction and cost-effectiveness using the Dutch Pediatric Quality of Life Healthcare Satisfaction and Institute for Medical Technology Assessment Productivity Cost Questionnaire.

**Results** Ninety-one (intervention: 54; control: 37) patients (56% boys) were included. Median (interquartile range) number of hospital visits was lower in the intervention group (1 vs 3; *p* < 0.001). All but one of the OSS patients (98%) were discharged home on the day of surgery. Postoperative complication (1.9% vs 2.7%; *p* = 0.787) and recurrence rates (0% vs 2.7%; *p* = 0.407) did not differ between the intervention and control patients. “General satisfaction,” “satisfaction with communication,” and “inclusion of family” were higher after OSS, while satisfaction about “information,” “technical skills,” and “emotional needs” were similar. Median (range) follow-up was 28 (15–36) months.

**Conclusions** Pediatric one-stop inguinal hernia repair seems to be an effective treatment strategy that limits the number of hospital visits and provides enhanced family satisfaction without compromising the quality of care.

## Introduction

Pediatric inguinal hernia is a surgical condition and for the optimal management of pediatric inguinal hernia repair, a tailored approach is recommended taking into consideration the local facilities, resources, and expertise of the medical team involved. 1 The Dutch organization of care requires children who need inguinal hernia repair to visit the hospital on at least three separate occasions: preoperative assessment by the pediatric surgeon, the anesthesiologist, and a third visit for surgical treatment. If we combine these visits into a so-called “one-stop-surgery” (OSS) program, together with the introduction of a properly organized previsit screening, preoperative visits will no longer be necessary and this consolidates confirmation of diagnosis, preoperative assessment, and surgical repair into a single hospital visit. 2 3

Successful introduction of an OSS program for children undergoing selected ambulatory procedures (e.g., circumcision or umbilical hernia repair) was first described over 20 years ago. 2 Thereafter, a limited number of colleagues showed the efficacious implementation of a pediatric OSS program and explored its effectiveness, financial benefits, improved patient and family experience, and enhanced institutional efficiency. 3 4 5 6 None of the authors previously compared these results to patients who received usual care. This study evaluates the efficiency, (cost-)effectiveness, and family satisfaction of pediatric OSS versus regular inguinal hernia repair.

## Materials and Methods

### Setting and Participants

Prospective observational study included children aged more than or equal to 3 months and American Society of Anesthesiologists (ASA) grades I–II (according to the ASA physical status classification system) with an inguinal hernia referred to Amsterdam UMC between March 2017 and December 2018. Exclusion criteria were: age less than 3 months and significant comorbidities (ASA grades III–IV; Table 1 ).

**Table 1:** Eligibility criteria for one-stop inguinal hernia repair

**Inclusion criteria**
Primary unilateral or bilateral inguinal hernia confirmed by GP or referring specialist
Age ≥ 3 months
ASA grades I or II
**Exclusion criteria**
Recurrent ipsilateral inguinal hernia
Age < 3 months or > 18 years
ASA grades III–V
High-risk disorders, e.g., moderate to severe sleep apnea, patients on oxygen, CPAP or BiPAP, and (congenital) heart disease

Abbreviations: ASA, American Society of Anesthesiologists; BiPAP, bi-level positive airway pressure; CPAP, continuous positive airway pressure; GP, general practitioner.

### One-Stop-Surgery Program

The OSS program started on June 8, 2017, in Amsterdam UMC, location VUmc: four OSS patients were scheduled daily as part of the regular day-care program with a total operation room capacity (and occupation) of eight cases per day. Parents/caretakers of eligible patients were offered OSS treatment by giving them access to a digital platform including a video that was especially designed to inform parents on their child's inguinal hernia (treatment). If they decided to participate, parents were able to schedule OSS treatment at a convenient day and received more detailed information about the appointments and preoperative nil by mouth instructions by email. Parents were asked to upload a picture of the swelling located in the groin(s) of their child to support inguinal hernia diagnosis. In OSS, patients first visit the outpatient clinic for physical examination and confirmation of diagnosis by the pediatric surgeon, followed by preoperative assessment by the anesthesiologist, after which the same surgeon will perform open inguinal hernia repair. All pediatric surgeons participated in the OSS. To minimize school and work absence, the OSS was scheduled on Thursday to allow children to recover over the weekend and return to school next Monday. In case OSS appointments remained unexploited until 1 week before OSS treatment, regular day-care surgery patients were scheduled instead. Following implementation of OSS for children with inguinal hernia, the program was repetitively evaluated with parents of patients and representatives of the “Child & Hospital Foundation” (a patient organization devoted to child medical care; Dutch: “Stichting Kind & Ziekenhuis”) to optimize OSS treatment before definitive start of the program.

### Usual Care

Parents who declined OSS treatment and eligible patients that already received part of conventional treatment (i.e., an ordinary outpatient clinic visit) were put on a waiting list for surgery and were included in the control group. No routine clinical follow-up visits were performed in both groups. Parents were instructed to return to the clinic in case of complications or development of a second, metachronous contralateral, or ipsilateral recurrent hernia.

### Legal Aspects of Informed Consent for Surgery

(Oral) informed consent for any treatment needs to be obtained by all legal representative(s) of the child, e.g., one or both parents or guardian(s) at any time before surgery. For children aged 11 years or younger, informed consent from parent(s)/guardian(s) is required for surgery. The child's oral consent is not compulsory, though it is obliged to provide information to the child about what is going to happen. For children aged between 12 and 16 years, both consent from their parent(s)/guardian(s) and the child's consent are obligatory before surgery. In case the child is 16 years or older, the child's consent is sufficient, provided that the child is mentally competent to decide for itself. For participation in medical trials, written informed consent from all legal representative(s) is required. 7

### Primary Outcome

Primary outcome was treatment efficiency, i.e., total number of hospital visits related to inguinal hernia (repair) and waiting time (days) between referral and surgery, assessed using prospectively collected data from electronic patient records.

### Secondary Outcomes

#### Effectiveness

Program effectiveness was evaluated in terms of perioperative complications during anesthesia and surgery (e.g., apnea, spermatic duct/vessel injury), postoperative complications within 3 weeks following surgery (e.g., bleeding/hematoma, hydrocele, wound infection), recurrence rate, and the percentage of patients that could be discharged home at the day of surgery. Complications were assessed using medical patient records and a questionnaire that was sent to parents 3 weeks after surgery. Electronic patient records were evaluated after a minimum follow-up of 1 year after surgery to assess development of a recurrent or contralateral hernia.

#### Family Satisfaction

Family satisfaction with health care provided to children with an inguinal hernia was evaluated using a modified version of the Dutch Pediatric Quality of Life (PedsQL) Healthcare Satisfaction Hematology/Oncology Specific Module. 8 This modified questionnaire was developed by an expert panel consisting of pediatric surgeons, researchers, and colleagues from the psychosocial department, and focused on day-care surgery. A digital version of the 27-item PedsQL satisfaction questionnaire was sent to parents using Castor Electronic Data Capture (Castor EDC) 1 week after discharge to assess family satisfaction on six subscales: “general satisfaction,” “information,” “inclusion of family,” “communication,” “technical skills,” and “emotional needs.” For each question, a 5-point Likert response scale was utilized (1 = very unsatisfied; 2 = unsatisfied; 3 = neutral, i.e., not unsatisfied and not satisfied; 4 = satisfied; 5 = very satisfied). The parent-reported items were transformed into a 0–100 scale (1 = 0; 2 = 25; 3 = 50; 4 = 75; 5 = 100); higher scores indicate higher family satisfaction. The Cronbach's *α* values in the present study were good for all subscales: “general satisfaction” (four items; *α* = 0.71), “information” (four items; *α* = 0.72), “inclusion of family” (three items; *α* = 0.85), “communication” (nine items; *α* = 0.84), “technical skills” (three items; *α* = 0.79), and “emotional needs” (four items; *α* = 0.81). Overall satisfaction and whether parents would recommend the treatment to friends/family were rated using a Visual Analog Scale ranging from 0 to 10.

#### Costs

Health care and indirect costs were measured using the Institute for Medical Technology Assessment Productivity Cost Questionnaire that was sent to parents 1 and 3 weeks after surgery, and valued using guideline prices recommended in the Netherlands Guideline for Economic Evaluations in Health Care (Netherlands Health Care Institute, Diemen, 2016). 9 Health care costs included all costs that were directly related to the intervention (e.g., outpatient clinic visits, hospital admission, surgical equipment). Costs for patient and family included travel expenses, expressed as transportation costs (calculated for number of hospital visits using an average distance from household to hospital/general practitioner × €0.19 per kilometer) and parking fees (€3 per visit). Costs in other sectors included productivity loss by parents/caretakers during work (presenteeism), due to work absence (absenteeism), and unpaid work. Work absenteeism was calculated per hour of lost revenue and determined using a median income.

### Statistical Analyses

Data were prospectively assembled using Castor EDC. Statistical analyses were based on the principle of intention-to-treat and performed using Statistical Package for the Social Sciences software, version 25.0.0.1 (IBM SPSS Statistics). Patient demographics and treatment characteristics are reported as median values with interquartile ranges (IQRs) for continuous, and as percentages for categorical variables. Independent samples *t* -test, Fisher's exact test, and Mann–Whitney *U* tests were used to compare groups. Scale scores from the PedsQL questionnaire were computed as the sum of items divided by the number of items answered. Cronbach's *α* was used to measure the internal validity of the PedsQL questionnaire, and α greater than 0.70 was considered high internal consistency. For analysis of family satisfaction, a *p* -value of *p* less than 0.008 was considered to be significant after using a Bonferroni correction for multiple testing on six different domains. The effect of OSS treatment on the different outcomes was analyzed with linear regression analyses. Besides crude analyses, also multiple linear regression analyses were performed to account for potential confounding factors.

## Results

**Fig. 1:**
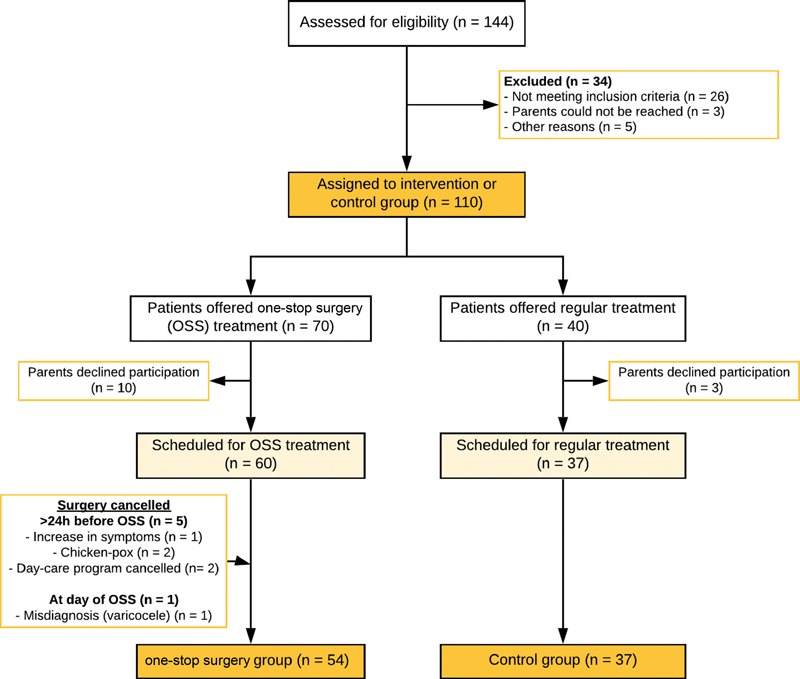
Flow diagram of patients included in this study.

Overall, 60 out of 110 eligible patients were scheduled for OSS treatment ( Fig. 1 ). In five patients, appointments were canceled more than 24 hours before the OSS because of a symptomatic hernia requiring earlier repair ( *n* = 1), chickenpox ( *n* = 2), and cancellation of the whole day-care program ( *n* = 2). In two patients, surgery was postponed and rescheduled beforehand due to illness and temporary absence of the hernia. In 1/58 (2%) OSS patients, a varicocele was diagnosed preoperatively and surgery was subsequently canceled. Thus, 54 and 37 patients were finally included in the intervention and control group, respectively.

### Patient Characteristics

Ninety-one patients (56% male) were included in this study. Control patients were younger compared with OSS patients—median age (IQR): 1 (0–3) year versus 5 (3–6) years; *p* less than 0.001. Sex and ASA classification did not differ between the groups. Most patients were referred by their general practitioner ( Table 2 ).

**Table 2:** Demographics of patients included in this study

	OSS ( *n* = 54)	Regular ( *n* = 37)	*p-* Value
**Sex**			0.541
Male, *n* (%)	30 (55.6)	21 (56.8)	
Female, *n* (%)	24 (44.4)	16 (43.2)	
**Age in years at hernia operation, median (IQR)**	5 (3–6)	1 (0–3)	< 0.001
**Hernia side,** ***n*** **(%)**			0.462
*Unilateral*	51 (94.4)	38 (97.3)	
* Right-sided*	26 (48.1)	23 (62.2)	
* Left-sided*	25 (46.3)	13 (35.1)	
*Bilateral*	3 (5.6)	1 (2.7)	
**ASA classification**			0.538
Grade I	48 (88.9)	31 (83.8)	
Grade II	6 (11.1)	6 (16.2)	
**Referred by,** ***n*** **(%)**			0.001
General practitioner	47 (87)	19 (51.4)	
Specialist in other hospital	4 (7.4)	11 (29.7)	
Specialist in same center	1 (1.9)	1 (2.7)	
Emergency department	2 (3.7)	6 (16.2)	

Abbreviations: ASA, American Society of Anesthesiologists; IQR, interquartile range; OSS, one-stop surgery.

### Primary Outcome

#### Efficiency

Time between referral and surgery (49 vs 55 days) was not significantly different between the OSS and control groups. OSS patients visited the hospital less often (1 vs 3 visits; *p* < 0.001).

### Secondary Outcomes

#### Effectiveness

In 29 (53.7%) OSS patients, preoperative hernia diagnosis was confirmed using an online picture of the swelling. There were zero no-shows in both groups. Fifty-three (98.1%) OSS patients were discharged at the day of surgery ( Table 3 ). One patient was discharged the next day due to postoperative pain following laparoscopic repair of a femoral hernia, after conversion from open to laparoscopic surgery since the hernia sac could not be identified. Operative and postoperative complication rates were similar between the OSS and control groups ( *p* = 0.787). Following OSS, one patient visited the emergency department because of a minor postoperative bleeding, requiring no intervention. In the control group also one patient visited the emergency department for postoperative fever without signs of wound infection. Development of contralateral and recurrent hernias was assessed after a median (range) follow-up time of 28 (15–36) months after surgery. One control patient versus none in the OSS group developed a recurrent hernia ( *p* = 0.407). Contralateral inguinal hernia occurred in two control (5.4%) and four OSS patients (7.4%).

**Table 3:** Treatment characteristics and complications

	OSS ( *n* = 54)	Regular ( *n* = 37)	*p* -Value
**Time in days from referral to surgery, median (IQR)**	49 (34.8–63.8)	55 (26.5–69)	0.987
**Total number of separate hospital visits, median (IQR)**	1 (1–1)	3 (2–3)	< 0.001
**Complications**			0.787
*Perioperative*			
Anesthetic problems	0 (0)	0 (0)	
Surgical complications	0 (0)	0 (0)	
*Postoperative complications/morbidity*			
Apnea	0 (0)	0 (0)	
Bleeding	1 (1.9)	–	
Fever (no signs of wound infection)	–	1 (2.7)	
** Discharged at same day after surgery, *n* (%) **	53 (98.1)	36 (97.3)	0.651
**Recurrent hernia**	0 (0)	1 (2.7)	0.407

Abbreviations: ASA, American Society of Anesthesiologists; IQR, interquartile range; OSS, one-stop surgery.

Results of linear regression analysis to estimate the effect of OSS treatment on primary (efficiency) and secondary (effectiveness) outcome parameters showed that OSS has a positive effect on the number of hospital visits. For the other outcomes, no significant results were observed ( Supplementary Table S1 ).

#### Family Satisfaction

**Fig. 2:**
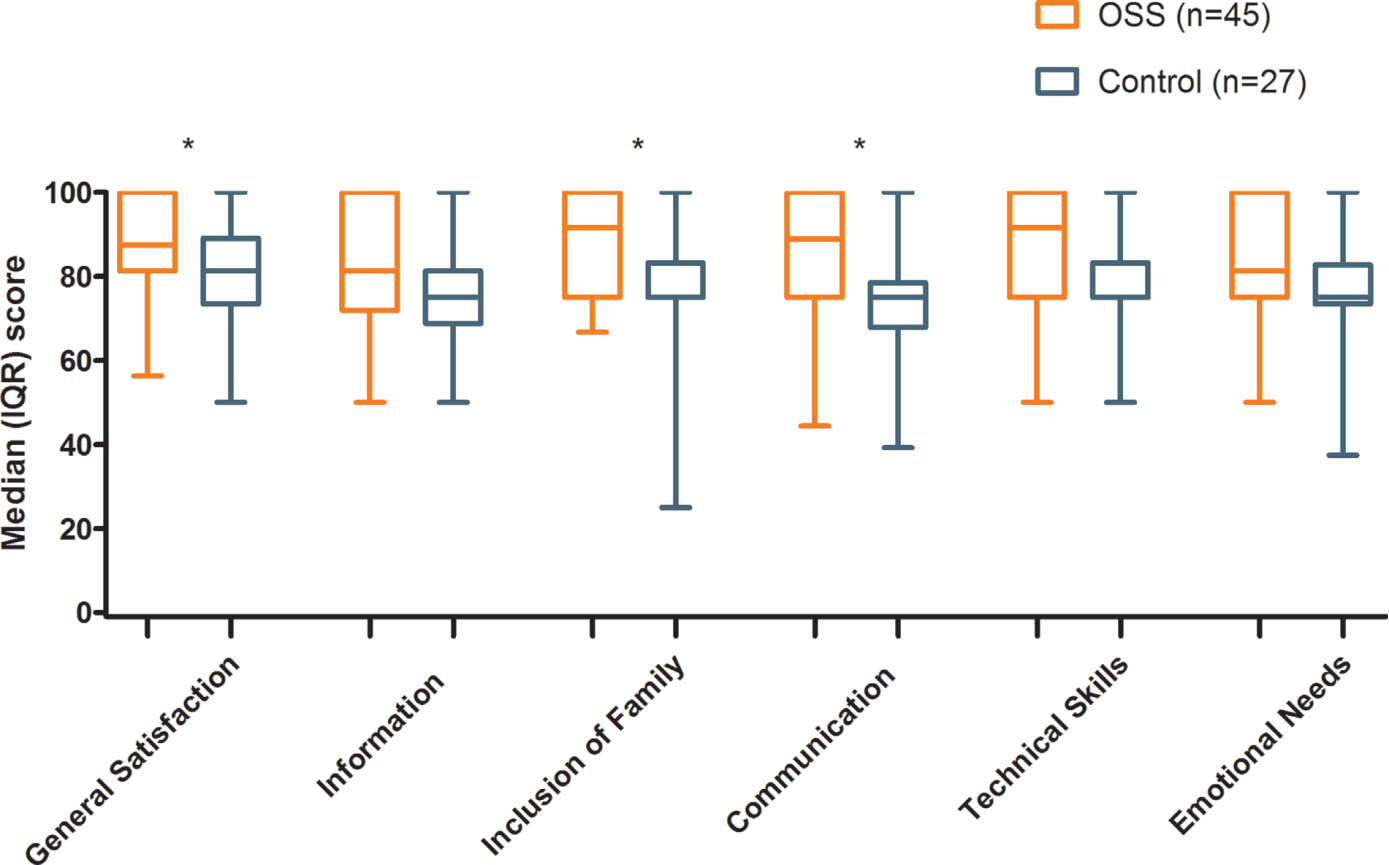
Results of the Dutch Pediatric Quality of Life Satisfaction questionnaire for one-stop surgery and control parents divided by six different subscales: “general satisfaction,” “information,” “inclusion of family,” “communication,” “technical skills,” and “emotional needs.”

PedsQL Healthcare Satisfaction questionnaire was completed by 83.3% of OSS and 73% of control families. Median (IQR) “general satisfaction” (87.5 (81.3–100) vs 81.3 (73.4–89.1), *p* = 0.007), satisfaction regarding “communication” (88.9 (75–100) vs 75 (67.9–78.5), *p* = 0.001), and “inclusion of family” (91.7 (75–100) vs 75 (75–83.3), *p* = 0.002) was higher after OSS ( Fig. 2 ). Satisfaction about “information” (81.3 (71.9–100) vs 75 (68.8–81.3), *p* = 0.113), “technical skills” (91.7 (75–100) vs 75 (75–83.3), *p* = 0.021), “emotional needs” (81.3 (75–100) vs 75 (73.4–82.8), *p* = 0.076), and overall satisfaction (mean ± standard deviation) was comparable between the OSS and control groups (8.6 ± 1.1 vs 8.2 ± 1.1, *p* = 0.15).

Results of linear regression analyses showed that OSS treatment has a positive effect on all subscales of the questionnaire ( Supplementary Table S2 ). Parents were more likely to recommend OSS treatment to family/friends compared with usual care (9.2 ± 1.3 vs 8.4 ± 1.8, *p* = 0.02).

#### Costs

Median total health care costs per OSS patient were lower compared with regular care (€2,112 vs €2,123, *p* < 0.001). Cost of work absenteeism was on average €245.65 versus €402.84 per parent of OSS and control families, respectively. Cost of unpaid work was reported by one OSS parent (€70) and three control parents (€700). Work productivity scores did not change across both OSS and control parents (mean value 7.6 vs 7.2, *p* = 0.368).

## Discussion

One-stop pediatric inguinal hernia treatment reduces the number of hospital visits and enhances parental satisfaction without reducing the quality and effectiveness of care. Ninety percent of the patients scheduled for OSS finally underwent OSS treatment, and the operation room utilization rate was 98%.

One of the prerequisites for a successful treatment program is that it should be restricted to diseases with a straightforward diagnosis, including a small number of false positive diagnoses, to prevent same-day cancellations. The inclusion of diseases should therefore be limited to diagnosis with a high likelihood of requiring surgery. Thereby, good preclinical screening is essential to identify eligible surgical candidates. As inguinal hernia is mostly a clinical diagnosis, implementation of eHealth technologies (e.g., obtaining an online picture) could improve its diagnostic accuracy.

One of the problems that was earlier encountered in OSS programs includes a high same-day cancellation rate, reported to be between 10 and 36%, mostly resulting from incorrect diagnosis ( Supplementary Table S3 ). 2 3 4 5 6 Precautionary measures to avoid cancellation resulting from misdiagnosis in the present study included that parents were asked to upload a picture of the swelling(s) prior to their hospital visit. Moreover, the Dutch health care system requires a general practitioner, pediatrician, or surgeon to refer patients to a secondary or tertiary care center, who had often already confirmed the diagnosis. Also, only patients with ASA grades I–II (mild systemic disease) were considered eligible for OSS treatment to minimize the risk of cancellation because of anesthetic contraindications.

Family satisfaction after OSS and regular care was previously evaluated by Olson et al; however, they could not demonstrate any group differences. 6 In this study, parental satisfaction, which can be used as a surrogate for treatment quality in low-risk procedures, was significantly different between the two groups. The major drivers of this difference were in the “inclusion of family” and “communication” domains, while satisfaction with “technical skills” was also largely increased after OSS. The implementation of eHealth technologies (e.g., possibility to use the digital platform with information on inguinal hernia and its treatment), a significant reduction in the number of hospital visits, and the ability to schedule the surgery themselves by choosing an appropriate date from a list of options are likely to be partly responsible for the enhanced satisfaction. Moreover, these results show the importance of personal contact and communication with parents of patients before surgery. To better prepare families for surgery and subsequently enhance future family satisfaction after conventional treatment, the methods we used for the intervention group have therefore been made accessible to all parents of children with inguinal hernia.

Last, the results of this study suggest that implementation of an OSS program has minor financial benefits from both a societal and institutional perspective. This is especially intriguing in the era of value-based health care; however, it should be noted that the marginal difference in health care costs is not expected to be clinically relevant, and the Dutch health care system cannot be generalized to other health care systems. OSS treatment is increasingly advantageous for families that have to travel a great distance from household to hospital since they only have to visit the hospital once. Moreover, introduction of an OSS together with the implementation of eHealth technologies to obtain correct diagnosis facilitates access to pediatric surgical care. Furthermore, we hypothesize that OSS treatment results in less school absence and potentially lessens the burden on preexistent waiting lists by eliminating the number of outpatient visits, and thereby allowing other patients an earlier visit.

This study has several limitations. Although this is the first study aimed at assessing the overall effect and impact before and after implementation of an OSS program on treatment efficiency, (cost-)effectiveness, and family satisfaction, it is a single-center observational study with a limited number of participants. Consequently, this could lead to selection bias, that might cause overestimation of family satisfaction since families choose to participate in the OSS, and potential confounding factors, that could have an influence on the outcome effect. Thereby, since the inclusion of diseases was restricted to diseases with a high likelihood of requiring surgery, the generalizability of the study results may be limited. Last, the survey response rate in OSS families was slightly higher, and families that opted to complete the survey could be expected to be more satisfied. At the same time, families that were not/less satisfied were potentially less likely to return the questionnaire, thereby increasing the risk for introducing nonresponsive bias, although both groups showed high family satisfaction.

## Conclusion

In conclusion, implementation of an OSS program is very promising as it seems effective and provides more efficient health care for children with inguinal hernia without compromising quality of care. Especially in light of the ongoing COVID-19 pandemic, reducing the number of (unnecessary) hospital visits is of great importance. OSS results in superior family satisfaction at lower costs, and a properly organized previsit screening may decrease the risk for false positive diagnoses and subsequent cancellations.

## Notes

**What Is Already Known on This Topic**

- Efficacious implementation of an OSS program for children undergoing selected ambulatory procedures has been proved to improve patient and family experience and institutional efficiency.
- One of the problems encountered in OSS programs includes a high same-day cancellation rate mostly resulting from incorrect diagnosis.
- The overall effect and impact before and after implementation of an OSS program on treatment efficiency, (cost-)effectiveness, and family satisfaction remains unquantified.

**What This Study Adds**

- One-stop pediatric inguinal hernia treatment provides more efficient health care for children with inguinal hernia without compromising the quality and effectiveness of care.
- Restriction to diseases with a high likelihood of requiring surgery and properly organized previsit screening diminish the risk for false positive diagnoses and subsequent cancellations.
- Implementation of OSS results in superior family satisfaction and highlights the importance of personal contact and pre- and perioperative communication with parents of patients.
