# Supplementary material for: One-Stop Surgery: An Innovation to Limit Hospital Visits in Children
Source: Eur J Pediatr Surg. 2021 Dec 2;32(5):435–42. doi: 10.1055/s-0041-1740158 (PMC9481276; doi:10.1055/s-0041-1740158)
Supplement: Supplementary file 1 — Supplementary Material [file 10-1055-s-0041-1740158-s215822oa.pdf]

Supplementary Table S1 Results of linear regression analyses to estimate the effect of one-stop surgery treatment on primary (efficiency) and secondary (effectiveness) outcome parameters

	Linear regression		
	B coefficient	SE B	*p*-Value
**Time from referral to surgery, days**			
Unadjusted	0.76	11.45	0.947
Adjusted[a]	0.16	13.39	0.990
**Total number of hospital visits**			
Unadjusted	−1.75	0.12	< 0.001
Adjusted[a]	−1.68	0.14	< 0.001
**Operative and postoperative complications**			
Unadjusted	−0.01	0.03	0.789
Adjusted[a]	−0.01	0.04	0.700
**Discharged at same day after surgery**			
Unadjusted	−0.01	0.03	0.789
Adjusted[a]	−0.00	0.04	0.985
**Recurrent hernia**			
Unadjusted	−0.03	0.02	0.229
Adjusted[a]	−0.04	0.03	0.125

Abbreviation: SE, standard error.
[a]Results adjusted for age and referral status.

Supplementary Table S2 Results of the linear regression analyses to estimate the effect of the one-stop surgery treatment on parental satisfaction

	Unadjusted linear regression			Adjusted[a] linear regression		
	B coefficient	SE B	*p*-Value	B coefficient	SE B	*p*-Value
**PedsQL Healthcare Satisfaction subscales**						
General satisfaction	7.97	3.04	0.011	8.38	3.46	0.018
Information	5.77	3.46	0.100	5.13	3.93	0.197
Inclusion of family	12.10	3.30	< 0.001	14.21	3.74	< 0.001
Communication	10.96	3.33	0.002	10.25	3.82	0.009
Technical skills	7.72	3.33	0.023	6.37	3.78	0.097
Emotional needs	7.46	3.68	0.046	7.77	4.19	0.068

Abbreviation: SE, standard error.
[a]Results adjusted for age and referral status.

 © 2021. The Author(s).

Supplementary Table S3 Day-of-surgery cancellation rates including the reasons for cancellation compared to previous one-stop surgery programs

			Reasons for cancellation					
**Author, year**	**Patients no.**	**Cancellation rate**	**Nil by mouth issues**	**Anesthetic contraindications**	**Incorrect diagnosis**	**Illness**	**No-show**	**Other reasons**
This study	63	2%	NA	NA	1	NA	NA	NA
Tagge et al 1999[2]	61	16%	NA	NA	NA	NA	9	1[a]
Barnett et al 2012[4]	61	18%	1	NA	5	4	NA	1[a]
Criss et al 2018[5]	45	36%	NA	2	NA	NA	NA	14[b]
Olson et al 2018[6]	90	19.5%	NA	NA	9	Unknown [c]	Unknown [c]	4[d]
Cunningham et al 2019[3]	89	10%	1	NA	6	NA	2	NA

Abbreviation: NA, not applicable.
[a]Parents would not proceed with surgical treatment or requested rescheduling of surgical treatment.
[b]Six diagnoses did not require surgical treatment and eight diagnoses opted conservative management.
[c]The remaining surgeries were cancelled because of both illness and no-show, though specific details regarding the exact numbers were missing.
[d]Three patients required further diagnostic testing; in one patient the procedure was rather performed in the clinic instead of the operating room.

 © 2021. The Author(s).
